# Crosslinking B-Cell Lymphoma (BCL1) in Surgery Patients by Exploring Its Therapeutic Potential for Head and Neck Cancer Pathology

**DOI:** 10.7759/cureus.82853

**Published:** 2025-04-23

**Authors:** Mostafa Ahmed Abdellah Ahmed, Amna Batool, Madeeha Minhas, Abdul Rehman Khalil Shaikh, Seemi Tanvir, Hafiz Muhammad Faizan Mughal, Muhammad Haseeb

**Affiliations:** 1 General Surgery, October 6th Hospital, 6th of October City, EGY; 2 Surgery, Fatima Memorial Hospital, Lahore, PAK; 3 Health Sciences, King Saud Bin Abdulaziz University for Health Sciences, Jeddah, SAU; 4 Pathology, Liaquat University of Medical and Health Sciences, Jamshoro, PAK; 5 Pathology, Margalla Institute of Health Sciences, Rawalpindi, PAK; 6 Internal Medicine, Khawaja Muhammad Safdar Medical College, Sialkot, PAK; 7 Oncology, Shaikh Zayed Hospital, Lahore, PAK

**Keywords:** bcl1, cancer biology, gene expression profiling, head and neck surgery, hodgkin lymphoma, squamous cell carcinoma

## Abstract

Background

Head and neck cancer (HNC) surrounds many malignancies that affect mucosal linings, lymphatic tissues, and salivary glands. The predominant subtypes include squamous cell carcinoma (SCC), Hodgkin lymphoma (HL), and pleomorphic adenoma (PA). One long non-coding RNA (lncRNA) known as B-cell lymphoma 1 (*BCL1)* has been observed to be a key regulator of tumor progression, metastasis, and resistance to chemotherapy.

Objective

This study aims to quantify the expression of *BCL1* across HNC subtypes to evaluate its diagnostic and prognostic relevance.

Materials and methodology

A case-control study was conducted for nine months from February 2023 to October 2023. The study involved 160 HNC patients and 40 healthy controls. Blood samples were collected, and RNA extraction, cDNA synthesis, and RT-qPCR analysis were done afterward using *BCL1-*specific primers. Data were analyzed by using one-way analysis of variance (ANOVA) in SPSS v.26 (IBM Corp, Armonk, NY, US) with p<0.05 considered statistically significant.

Results

In patients with HNC, elevated relative gene fold levels of *BCL1* highlighted malignancy in squamous cell carcinoma (SCC; 3.19±0.72), Hodgkin lymphoma (HL; 1.91±0.72), and pleomorphic adenoma (PA; 2.24±0.72), in comparison to the control group (1.07±0.72). SCC patients showed the highest expression, which correlated with advanced tumor stages (Stage IV: 60%).

Conclusion

There was an overexpression of *BCL1* observed in HNC subtypes, which highlighted its role as an important biomarker for tumor aggressiveness and therapeutic resistance. This advocates its integration into frameworks of precision oncology.

## Introduction

Head and neck cancer (HNC) comprises a range of malignancies such as the ones arising from the oral cavity, pharynx, larynx, sinuses, and salivary glands, which constitute upto 6% of global cancer cases [1]. Among them, squamous cell carcinoma is the most dominating, constituting over 90% of HNC cases, and it originates from the mucosal linings of the aerodigestive tract [2]. Major risk factors involve the use of tobacco, alcohol consumption, and infections caused by human papillomavirus (HPV), especially in oropharyngeal SCC [3]. Despite surgical and chemotherapeutic advances, SCC still is a challenge due to early nodal metastasis, resistance to drugs, and poor survival rate in advanced stages [4].

Beyond SCC, Hodgkin lymphoma (HL) and pleomorphic adenoma (PA) constitute those subtypes of HNC that are clinically distinct. HL is characterized by Reed-Sternberg cells and cervical lymphadenopathy, which is linked with Epstein-Barr virus (EBV)-caused infections and immune dysfunction [5]. While HL has a slightly more favorable prognosis with a possibility of early treatment, its rarity to be found in the head and neck region complicates its diagnosis [6]. Conversely, PA, the most common type of benign salivary gland tumor, is seen as a slow-growing mass, but it always carries the risk of malignancy, therefore necessitating vigilant monitoring [7].

Recent studies in molecular biology have highlighted the role of long non-coding RNAs (lncRNAs) in the pathogenesis of HNC. *BCL1 *has emerged as a critical controller of tumor progression, metastasis, and chemoresistance across a range of cancers [8]. In HNC, *BCL1* overexpression correlates with signs of malignancies such as epithelial-mesenchymal transition (EMT), lymph node metastasis, and poor survival [9,10].

This study aims to investigate *BCL1 *expression levels in HNC patients. It also aims to compare the SCC, HL, and PA subtypes with healthy controls. By illuminating its association with malignancy, this study aims to evaluate *BCL1’s* potential as a diagnostic biomarker and therapeutic target for strategies of precision oncology.

## Materials and methods

This six-month case-control study, from February 2023 to July 2023, enrolled 200 participants, of which 160 were HNC patients, including SCC (85%), HL (10%), and PA (5%), and 40 healthy controls. Inclusion criteria included individuals over 40 years of age, both male and female, with a confirmed HNC diagnosis and a histopathological record within the past 12 months. Inclusion criteria were age > 40 years, male or female, confirmed cases of HNC diagnosis with a histopathological history of 12 months, and healthy controls. Exclusion criteria were any metastatic cancer located outside the head and neck, lack of diagnostic reports or records, prior chemotherapy and radiotherapy, no clinical history, refusal to consent, and other malignancies. Based on the consecutive sampling technique, after informed consent, these participants were from tertiary care hospitals, mainly at Shaikh Zayed Hospital Lahore, October 6th Hospital, Egypt, and Fatima Memorial Hospital, Lahore, with Approval No. 23-1455, under the Declaration of Helsinki. Peripheral blood samples (5 mL) were collected using ethylenediaminetetraacetic acid (EDTA) tubes, transported on dry ice for 20 minutes, and stored at −80 °C until RNA was extracted. The total RNA was extracted and amplified by using the QIAamp Blood Kit (#51104, Qiagen, Hilden, Germany). The relative gene fold was calculated via the ΔΔCt method, and GAPHD was used as the internal control (IC). The *BCL1*-specific primers are shown in Table 1. One-way analysis of variance (ANOVA) was utilized to check comparative analysis among samples with IBM SPSS statistics version 26.0 (IBM Corp, Armonk, NY, US) with a p-value of <0.05 as statistically significant.

**Table 1:** Targeted primer sets

Gene Name	Primer Sequences
BCL1	Forward: 5’ GCGCAGCGCCATTTTAGCCA 3’
	Reverse: 5’ GAGTGGCTGAGAGGGCTTTT 3’

## Results

The study consisted of 200 participants, who were divided into 160 HNC patients and 40 healthy controls. The male-to-female ratio was 3:1 among patients (mean age 48.2 ± 12.5 years), and gender distribution was balanced in the controls (mean age 45.6 ± 11.8 years). Family history was observed in 75% of patients. SCC accounted for 85% of cases (n = 136), followed by HL (10%; n = 16), and PA (5%; n = 8). SCC predominated in advanced-stage disease, Stage III (40%) and Stage IV (60%), showing an aggressive clinical presentation (Table 2).

**Table 2:** Summary of study characteristics

Variable	Patients (n=160)	Controls (n=40)
Age (Mean ± SD)	48.2 ± 12.5	45.6 ± 11.8
Family History (%)	75	0
Tumor Stage III (%)	40	N/A
Tumor Stage IV (%)	60	N/A

**Figure 1:**
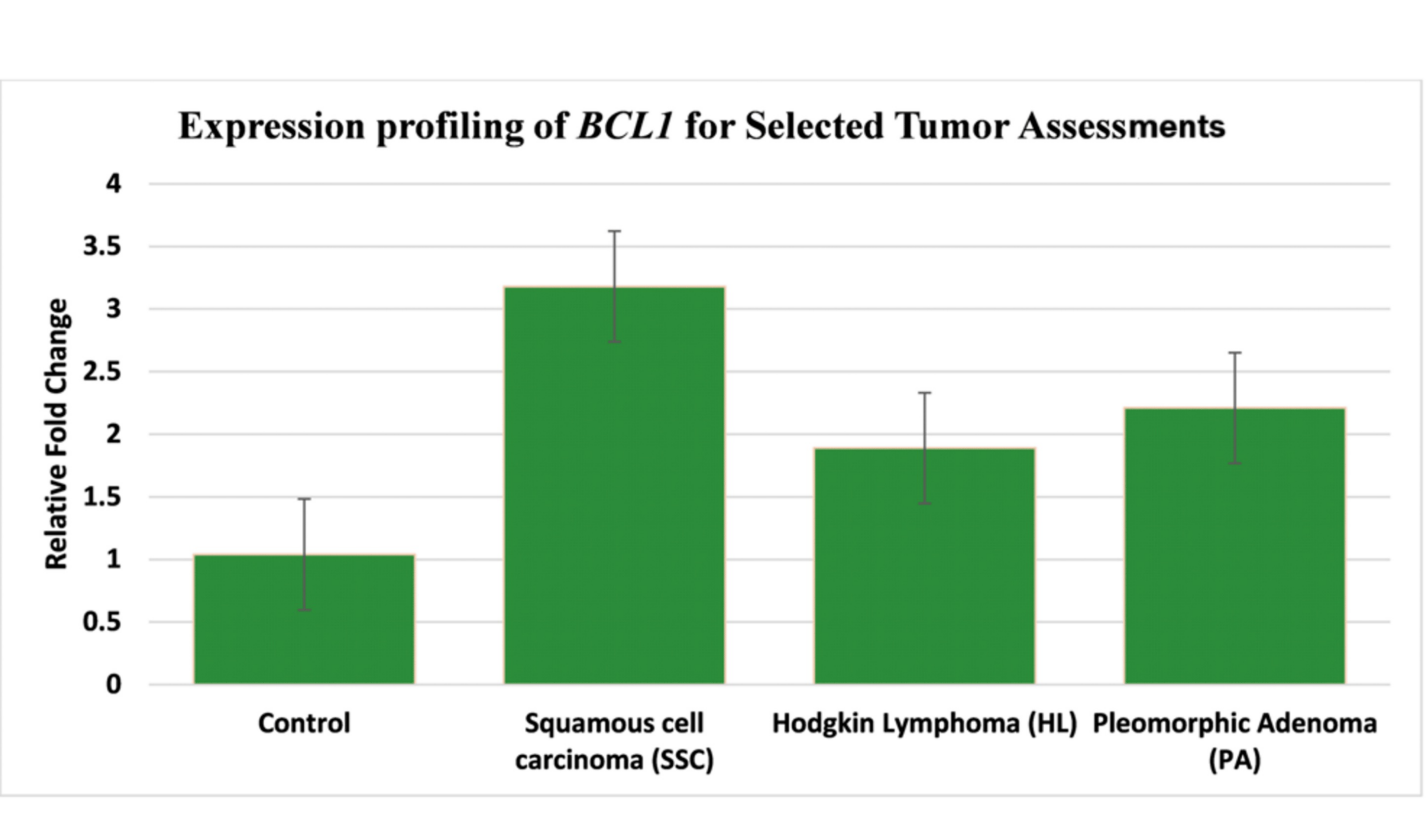
Relative gene fold calculations

The finding of quantitative reverse transcription polymerase chain reaction (RT-qPCR) demonstrated significant overexpression of *BCL1 *across all subtypes of HNC in comparison with controls (p<0.05). SCC revealed the highest expression (3.19 ± 0.72-fold), next was PA (2.24 ± 0.72-fold), and finally, HL showed the least rise in expression (1.89 ± 0.72-fold), against a threshold of 1.07 ± 0.72-fold in controls (Figure 1). One-way ANOVA showed intergroup variability (p < 0.001), demonstrating noticeable differences between SCC and controls (p < 0.001), SCC and HL (p = 0.008), and SCC and PA (p = 0.021). HL and PA also showed clear differences from controls (p = 0.015 and p = 0.009, respectively).

There was progressive *BCL1* upregulation in SCC between Stage IV tumors (> 3.89 ± 0.65-fold) and Stage III tumors (2.91 ± 0.58-fold; p = 0.003), as stratified by tumor stage during subgroup analyses. Levels of* BCL1* in PA showed similar trends as seen in early malignant transformations (controls vs. 2.24 ± 0.72 vs. PA, p = 0.009).

## Discussion

Investigations from this study highlighted the elevated expression of *BCL1 *across three HNC subtypes, i.e., SCC, HL, and PA, revealing a significant correlation in all subtypes in comparison to healthy controls. These findings align with the emerging evidence that *BCL1*, a gene expression and chromatin modeling regulator, plays a significant role in the progression of tumors, metastasis, and therapeutic resistance across various types of malignancies, mainly solid and hematological ones [11]. In SCC, *BCL1* expression was significantly elevated (3.19-fold). These results were consistent with *BCL1’s *relation with epithelial-mesenchymal transition (EMT), a process that is critical for invasion of the tumor and lymph node metastasis [12]. EMT is defined by the loss of epithelial markers and gain of mesenchymal markers such as E-cadherin and vimentin, respectively. These markers enable cancer cells to detach from their primary site and spread [13]. Preclinical studies demonstrated that *BCL1 *silencing transposes EMT, reduces the volume of the tumor, and increases chemosensitivity in SCC models. This suggested its direct involvement in these pathways [14].

HL is rarely found in the head and neck region, but when it occurs in this region, it exhibits a significant increase in *BCL1* expression (1.89-fold). This finding aligns with recent reports that linked *BCL1* to NF-κB signaling and Reed-Sternberg cell proliferation, both of which are mechanisms central to HL pathogenesis. While HL prognosis is generally favorable with early therapies, refractory cases remain challenging [15,16]. Notably, *BCL1* overexpression was also seen in PA (2.24-fold), a benign tumor of the salivary gland. While PA lacks the potential for metastasis like SCC, its ability to recur and malignant transformation necessitates the use of biomarkers for early detection. Studies on malignant PA revealed that dysregulation in *BCL1* aids in malignant changes, potentially via interactions with PLAG1 oncogenes or disruption of salivary gland differentiation pathways [17]. These findings highlight *BCL1’s* potential both as a biomarker for risk stratification and as a therapeutic agent. In SCC, activation of *BCL1*-driven pathways, such as P13K/AKT, suggests that antisense oligonucleotides or small-molecule inhibitors can improve existing therapies, particularly in cisplatin-resistant cases [18].

Future studies should perform multicenter collaborations, which would enable subgroup analyses by tumor stage, HPV status, and treatment history. In vitro knockdown models or patient-derived xenografts would be beneficial for the validation of underlying functional mechanisms. Prospective longitudinal studies would help track *BCL1* levels both before and after the treatment, which would help in disease monitoring after treatment and the efficacy of treatment.

## Conclusions

This study identified *BCL1* as an overexpressed and dysregulated lncRNA in head and neck cancer subtypes, which implies its ability in diagnosis, prognosis, and therapeutic targeting. Its elevation in SCC, HL, and PA highlighted both common as well as distinct pathogenic mechanisms that reflected the heterogeneity of HNC. While SCC remained the prime candidate for BCL1-directed therapies, the findings of HL and PA opened new avenues for research in the fields of lymphomagenesis and benign to malignant transformation. Future work should focus on prioritizing studies on mechanistic and clinical validation, which would improve therapeutic efficacy and, ultimately, patient outcomes.
